# Status epilepticus in patients with brain tumors and metastases: A multicenter cohort study of 208 patients and literature review

**DOI:** 10.1186/s42466-024-00314-7

**Published:** 2024-04-04

**Authors:** Johanna K. Rickel, Daria Zeeb, Susanne Knake, Hans Urban, Jürgen Konczalla, Katharina J. Weber, Pia S. Zeiner, Axel Pagenstecher, Elke Hattingen, André Kemmling, Emmanouil Fokas, Sebastian Adeberg, Robert Wolff, Martin Sebastian, Tillmann Rusch, Michael W. Ronellenfitsch, Katja Menzler, Lena Habermehl, Leona Möller, Marcus Czabanka, Christopher Nimsky, Lars Timmermann, Christian Grefkes, Joachim P. Steinbach, Felix Rosenow, Leena Kämppi, Adam Strzelczyk

**Affiliations:** 1 Epilepsy Center Frankfurt Rhine-Main, Department of Neurology, Goethe-University and University Hospital Frankfurt, Schleusenweg 2-16, 60528 Frankfurt, Germany; 2 Center for Personalized Translational Epilepsy Research (CePTER), Goethe-University Frankfurt, Frankfurt, Germany; 3 Department of Neurology and Epilepsy Center Hessen, Philipps-University Marburg, Marburg, Germany; 4 Department of Neurosurgery, Philipps-University Marburg, Marburg, Germany; 5 Dr Senckenberg Institute of Neurooncology, University Hospital and Goethe-University Frankfurt, Frankfurt, Germany; 6 Department of Neurosurgery, Goethe-University Frankfurt, Frankfurt, Germany; 7 Frankturt Cancer Institute (FCI), Goethe-University Frankfurt, Frankfurt, Germany; 8 German Cancer Research Center (DKFZ) Heidelberg, Germany and German Cancer Consortium (DKTK), Partner Site Frankfurt/Mainz, Frankfurt, Germany; 9 Institute of Neurology (Edinger-Institute), Goethe-University Frankfurt, Frankfurt, Germany; 10 Institute of Neuropathology, Philipps-University Marburg, Marburg, Germany; 11 Institute of Neuroradiology, Goethe-University Frankfurt, Frankfurt, Germany; 12 Department of Neuroradiology, Philipps-University Marburg, Marburg, Germany; 13 Department of Radiotherapy and Oncology, Goethe-University Frankfurt, Frankfurt, Germany; 14 Department of Radiation Oncology, UKGM Marburg, Marburg, Germany; 15 Marburg Ion-Beam Therapy Center (MIT), Department of Radiation Oncology, UKGM Marburg, Marburg, Germany; 16 Gamma Knife Frankfurt, Frankfurt, Germany; 17 Hematology/Oncology, Department of Medicine II, University Hospital Frankfurt, Frankfurt, Germany; 18 Department of Hematology, Oncology & Immunology, Philipps-University Marburg, Marburg, Germany; 19 Epilepsia Helsinki, European Reference Network EpiCARE, Department of Neurology, Helsinki University Hospital and University of Helsinki, Helsinki, Finland; 20 University Cancer Center (UCT) Frankfurt-Marburg, Frankfurt, Marburg, Germany

**Keywords:** Glioblastoma, Astrocytoma, Meningioma, Epilepsy, Seizure

## Abstract

**Objective:**

Brain tumors and metastases account for approximately 10% of all status epilepticus (SE) cases. This study described the clinical characteristics, treatment, and short- and long-term outcomes of this population.

**Methods:**

This retrospective, multi-center cohort study analyzed all brain tumor patients treated for SE at the university hospitals of Frankfurt and Marburg between 2011 and 2017.

**Results:**

The 208 patients (mean 61.5 ± 14.7 years of age; 51% male) presented with adult-type diffuse gliomas (55.8%), metastatic entities (25.5%), intracranial extradural tumors (14.4%), or other tumors (4.3%). The radiological criteria for tumor progression were evidenced in 128 (61.5%) patients, while 57 (27.4%) were newly diagnosed with tumor at admission and 113 (54.3%) had refractory SE. The mean hospital length of stay (LOS) was 14.8 days (median 12.0, range 1–57), 171 (82.2%) patients required intensive care (mean LOS 8.9 days, median 5, range 1–46), and 44 (21.2%) were administered mechanical ventilation. All patients exhibited significant functional status decline (modified Rankin Scale) post-SE at discharge (p < 0.001). Mortality at discharge was 17.3% (n = 36), with the greatest occurring in patients with metastatic disease (26.4%, p = 0.031) and those that met the radiological criteria for tumor progression (25%, p < 0.001). Long-term mortality at one year (65.9%) was highest in those diagnosed with adult-type diffuse gliomas (68.1%) and metastatic disease (79.2%). Refractory status epilepticus cases showed lower survival rates than non-refractory SE patients (log-rank p = 0.02) and those with signs of tumor progression (log-rank p = 0.001).

**Conclusions:**

SE occurrence contributed to a decline in functional status in all cases, regardless of tumor type, tumor progression status, and SE refractoriness, while long-term mortality was increased in those with malignant tumor entities, tumor progressions, and refractory SE. SE prevention may preserve functional status and improve survival in individuals with brain tumors.

**Supplementary Information:**

The online version contains supplementary material available at 10.1186/s42466-024-00314-7.

## Introduction

Epilepsy is a frequent symptom of patients with brain tumors, which are strong risk factors for seizures (relative risk = 40) [1] despite representing only 4% of all epilepsy etiologies [1–3]. In addition, greater than one-third of patients with brain tumors develop epilepsy [4, 5]. Seizures are the presenting clinical signs of tumors in 30–50% of patients, and an additional 10–30% exhibit epilepsy later in the disease course, often in association with neurosurgical procedures or disease progression [1, 4–8]. The prevalence of epilepsy varies greatly according to tumor type and location and appears to be inversely proportional to tumor grade, with reported frequencies approximating 100% in non-malignant dysembryoblastic neuroepithelial tumors, 29–49% in high-grade gliomas, 20–35% in metastases, and 10% in primary central nervous system (CNS) lymphomas [6, 9].

Status epilepticus (SE) is one of the most common neurological emergencies [10], which according to the latest definition proposed by the International League Against Epilepsy (ILAE), is a condition resulting from either the activation of abnormal mechanisms that prolong seizures or the failure of mechanisms that terminate seizure activity. SE can lead to cell damage, neuronal death, neuronal injury, or alteration of neuronal networks, depending on seizure type. Therefore, prompt seizure termination and prevention of recurrence is crucial in these cases [11–13].

The reported proportion of patients with SE that is attributable to brain tumors is 3–12% [10, 14]. However, limited information is available that specifically evaluates SE in this population [15]. Although seizures generally occur in low-grade malignancies and early disease stages, SE is more commonly found in high-grade tumors and later stages, suggesting a potentially distinct pathophysiological mechanism [9, 16]. A systematic review reported a higher short-term mortality in tumor-related SE cases compared to SE patients with other etiologies, although that study provided insufficient data to assess differences in treatment response, long-term mortality, or morbidity [15]. Two independent reviews indicated that tumor-associated SE (TASE) had a distinct clinical profile and stressed the need for prospective multi-center studies [9, 15].

The aim of this study was to use a large multi-center cohort to systematically describe the clinical characteristics, treatment, and short- and long-term outcomes of patients presenting with SE secondary to primary brain tumors and brain metastases.

## Material and methods

### Study design and setting

This retrospective, multicenter cohort study was conducted at the university hospitals of Frankfurt and Marburg between January 2011 and December 2017. Both hospitals offered a full range of neurological care services with expertise in epileptology, neurooncology, and intensive care medicine. Frankfurt University Hospital serves primarily an urban area[17], while its Marburg counterpart possesses a neurological department that services the city and surrounding rural areas, managing a population of more than 500,000. Due to its representative population structure, the area surrounding Marburg has been used for a population-based estimate of the incidence of SE in Germany [18].

This study included all adult patients (aged 18 or older) admitted to hospital due to non-anoxic SE with brain tumors or metastases. Only the first episode of SE during the study period was analyzed. Patient records were reviewed and data were collected using standardized study forms and ascertained by the study coordinator (AS) and one independent researcher at each site (SK, FR). The study was approved by local ethics committees and adhered to the STROBE guidelines [19].

### Definition of SE

Following the latest definition and classification of SE published by the International League Against Epilepsy (ILAE) task force in 2015, all tonic–clonic seizures lasting > 5 min and focal seizures with impaired consciousness lasting > 10 min were considered SE events [11]. Refractory status epilepticus (RSE) is defined as recurrent seizure activity after two appropriately selected and dosed antiseizure medications (ASMs), which include a benzodiazepine. Super‐refractory status epilepticus (SRSE) is an SE that continues or recurs ≥ 24 h after the initiation of treatment with anesthetic drugs and includes cases in which seizure control is attained after induction of anesthesia but recurs once the anesthetic agent is tapered off [10, 20–22]. Nonconvulsive SE (NCSE) is defined according to the Salzburg criteria [23].

### Definition of measures

The patients were divided into four subgroups based on tumor category: adult-type diffuse gliomas, including glioblastomas and other CNS WHO Grade 2–4 gliomas; intracranial extradural tumors, including meningiomas and other benign extradural tumors; metastatic entities, including solid metastases and leptomeningeal metastatic diseases; and other tumors, such as CNS lymphomas. The latter group was not included in the analysis at the subgroup level due to the small number and heterogeneity of the cases. In addition, patients were separated into two groups based on the presence of progression signs. Those who displayed the neuroradiological criteria for tumor growth, tumor recurrence, increased perifocal edema, or imaging changes (i.e., pseudoprogression) due to radiation therapy and/or systemic chemotherapy were classified into the group with tumor progression signs.

Data regarding patient and SE characteristics, as well as the treatment during the SE, tumor treatment prior to the SE, and patient outcomes, were collected. Comorbidities of the patients were scored using the Charlson Comorbidity Index (CCI) [24]. The functional status of the patients was determined using a modified Rankin scale (mRS) at admission and discharge (0–2 = good functional status, 3–5 = poor functional status, 6 = deceased) [25]. SE severity was evaluated using the SE severity score (STESS) [26] and the level of refractoriness (non-refractory = NRSE, refractory = RSE, super-refractory = SRSE). The short-term outcomes of the patients were defined using mortality, functional status (mRS), and destination at hospital discharge, while long-term outcomes were considered as mortality within one-year post-discharge.

### Statistics

Statistical analyses were performed using IBM SPSS Statistics Version 28.0. Descriptive data are presented as minimum, maximum, median, mean ± standard deviation (SD), range, and percentage values. Univariable comparisons of proportions were calculated using Pearson's chi-squared test. The Mann–Whitney U test was applied for comparisons of variables of ordinal or non-normally distributed data. Long-term outcomes were depicted using Kaplan–Meier survival curves, and the log-rank test was performed for comparisons between the subgroups. Two-sided p-values of less than 0.05 were considered to be significant in all statistical analyses.

## Results

During the study period, 208 patients presenting with SE due to a brain tumor or metastasis were identified. The mean age was 61.5 ± 14.7 years (median 63 years; range 23–89 years) and 106 patients (51%) were male. Adult-type diffuse gliomas formed the largest subgroup with 116 (55.8%) patients, with glioblastoma as the most common tumor type (n = 85, 40.9%), followed by intracranial extradural tumors (30 cases, 14.4%) and metastatic entities (53 cases, 25.5%). Details regarding tumor entities are presented in Table 1, and localization data are listed in Additional file 1: Table S1. At admission, 128 (61.5%) patients presented with the neuroradiological criteria for tumor progression, while the remaining 80 (38.5%) had no signs of progression in the imaging.

**Table 1:** Primary brain tumor and brain metastasis characteristics of patients presenting with SE (n = 208)

Variable	N (%)
All patients	208 (100)
Adult-type diffuse gliomas	116 (55.8)
Glioblastoma	85 (40.9)
Other	31 (14.9)
Anaplastic astrocytoma	18 (8.7)
Oligoastrocytoma	7 (3.4)
Diffuse astrocytoma	5 (2.4)
Gliomatosis cerebri	1 (0.5)
Intracranial extradural tumors	30 (14.4)
Meningioma	26 (12.5)
Other	4 (2.0)
Acoustic neuroma	1 (0.5)
Adamantinous craniopharyngioma	1 (0.5)
Prolaktinoma	1 (0.5)
Pineal tumor	1 (0.5)
Metastatic entities (primary tumors)	53 (25.5)
Solid Metastasis	47 (22.6)
Lung cancer (NSCLC & SCLC)	19 (9.1)
Malignant melanoma	10 (4.8)
Breast cancer	6 (2.9)
Colon adenocarcinoma	5 (2.4)
Urothelial carcinoma	1 (0.5)
Pharyngeal carcinoma	1 (0.5)
Epitheloid angiosarcoma	1 (0.5)
Renal cell carcinoma	1 (0.5)
Uterine carcinoma	1 (0.5)
Prostate cancer	1 (0.5)
CUP	1 (0.5)
Leptomeningeal metastatic disease	6 (2.9)
Lung cancer (NSCLC & SCLC)	2 (1.0)
Testicular teratocarcinoma	1 (0.5)
Urothelial carcinoma	1 (0.5)
Cutaneous t-cell lymphoma	1 (0.5)
Esophageal carcinoma	1 (0.5)
Other	9 (4.4)
Lymphoma	7 (3.4)
PNET	1 (0.5)
Other	1 (0.5)

*NSCLC* Non-small-cell lung cancer, *SCLC* Small-cell lung cancer, *CUP* Cancer of Unknown Primary, *PNET* Primitive Neuro-Ectodermal Tumor

### Clinical characteristics of the SE patients

Table 2 shows the patient characteristics, SE characteristics, and patient outcomes for all individuals, as well as the univariate subgroup analysis according to tumor category and tumor progression signs at admission.

**Table 2:** Patient characteristics, status epilepticus characteristics, and outcomes stratified by tumor categories and tumor progression signs

Variable	All Cases	Tumor category*				Tumor progression signs		
		Adult-type diffuse gliomas	Intracranial extradural tumors	Metastatic entities	p-value	Yes	No	p-value
	N (%)	N (%)	N (%)	N (%)		N (%)	N (%)	
All cases	208 (100)	116 (55.8)	30 (14.4)	53 (25.5)		128 (61.5)	80 (38.5)	
Patient Characteristics								
Age at SE onset (years)								
Mean ± SD	61.5 ± 14.7	59.1 ± 15.4	70.8 ± 12.8	60.9 ± 11.6	& < 0.001; #0.003; §n.s	61.4 ± 15.4	61.5 ± 13.6	n.s
Median (Range)	62.5 (23–89)	60.5 (23–88)	72.5 (33–89)	62 (31–96)		63.5 (23–89)	61 (31–88)	
Sex								
Male	106 (51)	72 (62)	7 (23.3)	22 (41.5)	& < 0.001; #0.046; §0.009	66 (51.6)	40 (50)	n.s
Charlson comorbidity Index (CCI)								
2–5	145 (69.7)	110 (94.8)	26 (86.7)	0	&n.s.; # < 0.001; § < 0.001	81 (63.3)	64 (80)	0.008
> 6	63 (30.3)	6 (5.2)	4 (13.3)	53(100)		47 (36.7)	16 (20)	
Previous history of seizures								
Yes	127 (61.1)	83 (71.6)	17 (56.7)	23 (43.4)	&0.045; #n.s.; § < 0.001	69 (53.9)	58 (72.5)	0.007
Previous history of SE								
Yes	22 (10.6)	14 (12)	5 (16.7)	3 (5.7)	n.s	12 (9.4)	10 (12.5)	n.s
mRS before admission								
0–2	118 (56.7)	66 (56.9)	18 (60)	27 (50.9)	n.s	69 (53.9)	49 (61.3)	n.s
3–5	90 (43.3)	50 (43.1)	12 (40)	26 (49.1)		59 (46.1)	31 (38.8)	
SE Characteristics								
STESS								
0–3	176 (84.6)	104 (89.7)	18 (60)	46 (86.8)	& < 0.001; #0.002; §n.s	110 (85.9)	66 (82.5)	n.s
4–6	32 (15.4)	12 (10.3)	12 (40)	7 (13.2)		18 (14.1)	14 (17.5)	
Refractoriness of SE								
NRSE	95 (45.7)	57 (49.1)	10 (33.3)	24 (45.3)	n.s. **	56 (43.8)	39 (48.8)	n.s.**
RSE	94 (45.2)	50 (43.1)	15 (50)	25 (47.2)		61 (47.7)	33 (41.3)	
SRSE	19 (9.1)	9 (0.8)	5 (16.7)	4 (7.5)		11 (8.6)	8 (10)	
Tumor progression signs								
Yes	128 (61.5)	71 (61.2)	9 (30)	41 (77.4)	& < 0.001; # < 0.001; §0.05	128 (100)	0	n.t
Tumor diagnosed at admission								
Yes	57 (27.4)	22 (19)	8 (26.7)	23 (43.4)	&n.s.; #n.s.; §0.001	53 (41.4)	4 (5)	< 0.001
Time between tumor diagnosis and SE onset (years)								
Mean ± SD	2.3 ± 4.4	2.3 ± 4.1	5.3 ± 7.1	0.6 ± 0.9	&0.003; #0.002; §n.s	1.7 ± 4.0	3.2 ± 4.9	< 0.001
Median (Range)	0.5 (0–24.5)	0.6 (0–24.5)	2.1 (0–21.2)	0.2 (0–4.5)		0.3 (0–24.5)	1.2 (0–21.2)	
Outcome								
mRS at discharge								
0–2	61 (29.4)	36 (31)	8 (26.7)	14 (26.4)	n.s	28 (21.9)	33 (41.3)	0.003
3–6	147 (70.7)	80 (69)	22 (73.3)	39 (73.6)		100 (78.1)	47 (58.8)	
Discharge destination								
Home	79 (38.0)	49 (42.2)	7 (23.3)	19 (35.8)		46 (36)	33 (41.3)	
Rehabilitation	39 (18.8)	21 (18.1)	14 (46.7)	4 (7.5)		16 (12.5)	23 (28.8)	
Other hospital	21 (10.1)	9 (7.8)	3 (10)	7 (13.2)		12 (9.4)	9 (11.3)	
Nursing home	9 (4.3)	5 (4.3)	1 (3.3)	3 (5.7)		6 (4.7)	3 (3.8)	
Palliative Care/Hospice	15 (7.2)	10 (8.6)	0	4 (7.5)		12 (9.4)	3 (3.8)	
Other	9 (4.3)	7 (6)	0	2 (3.8)		4 (3.1)	5 (6.3)	
Mortality								
In-hospital	36 (17.3)	15 (13)	5 (16.7)	14 (26.4)	&n.s.; #n.s.; §0.031 ***	32 (25)	4 (5)	< 0.001 ***
30 days	50 (24.0)	26 (22.4)	5 (16.7)	17 (32.1)	n.s	41 (32)	9 (11.2)	0.001 ***
1 year	137 (65.9)	79 (68.1)	11 (36.7)	42 (79.2)	&0.003; # < 0.001; §n.s. ***	98 (76.5)	39 (48.8)	< 0.001 ***

p-values as pair-wise comparisons between tumor categories: & Adult-type diffuse gliomas vs Intracranial extradural tumors; # Intracranial extradural tumors vs Metastatic entities; § Adult-type diffuse gliomas vs Metastatic entities; * Tumor group Other is not presented; ** Comparison between NRSE and RSE/SRSE; *** Comparison between deceased and living

#### Patient characteristics

Patients with intracranial extradural tumors were significantly older than those in other tumor categories. The sex distribution showed higher proportions of female patients with intracranial extradural tumors (n = 23, 76.6%) and male patients with adult-type diffuse gliomas (n = 72, 62%). High comorbidity loads (CCI ≥ 6), were detected in 63 (30.3%) patients, most of whom had metastatic entities (n = 53, 84.1%), whereas patients in the other tumor subgroups generally had significantly lower CCI scores (2–5). The majority of patients (n = 127, 61.1%) had a previous history of seizures, and this finding was more pronounced among patients with no signs of tumor progression (n = 58, 72.5%) or adult-type diffuse gliomas (n = 83, 71.6%) at admission. In contrast, SE was often the first epileptic manifestation in patients with metastatic entities compared with those in other categories. The mRS before the SE showed no significant differences between the subgroups.

#### SE characteristics

A favorable STESS score of 0–3 points was present in 176 (84.6%) patients at admission, and this fraction was significantly lower in intracranial extradural tumor patients (n = 18, 60%), who tended to exhibit lower proportions of NRSE cases (n = 10, 33.3% vs. all patients n = 95, 45.7%) and higher proportions of SRSE cases (n = 5, 16.7% vs. all patients n = 19, 9.1%). Tumor progression signs (n = 128, 61.5%) were common in patients with metastatic entities (n = 41, 77.4%) and less prevalent in those with intracranial extradural tumors (n = 9, 30%). Brain tumors were newly diagnosed at SE admission in 57 patients (27.4%) and more frequently in patients with metastatic entities (n = 23, 43.3%) and tumors with radiological progression (n = 53, 41.4%) than those with adult-type diffuse gliomas (n = 22, 19%) and tumors without radiological progression (n = 4, 5%). The time between tumor diagnosis and SE averaged 2.3 ± 4.4 years (median 0.5, range 0–24.5 years), with the shortest time interval occurring for the metastatic entities (mean 0.6 ± 0.9, median 0.2, range 0–4.5 years) and the longest for the intracranial extradural tumor group (mean 5.3 ± 7.1, median 2.1, range 0–21.2 years).

### Treatment

On admission, 61 (29.3%) patients were receiving ongoing tumor-specific therapy and 53 (25.5%) experienced the SE within six months of completing radiation therapy. Patients with adult-type diffuse gliomas had significantly more ongoing treatment (n = 44, 37.9%) and radiation therapy in the six-month period (n = 43, 37.1%) than those in the other groups; details are presented in Table 3. None of the patients with intracranial extradural tumors had ongoing therapy or radiation therapy close to the time of the SE event.

**Table 3:** Hospital treatment, medication used for SE treatment, and tumor treatments prior to and during SE, stratified by tumor categories and tumor progression signs

Variable	All Cases	Tumor category				Tumor progression signs		
		Adult-type diffuse gliomas	Intracranial extradural tumors	Metastatic entities	p-value	Yes	No	p -value
	N (%)					N (%)	N (%)	
All Cases	208(100)	116 (55.8)	30 (14.4)	53 (25.5)		128 (61.5)	80 (38.5)	
Hospital treatment								
Length of hospital stay (days)								
Mean ± SD	14.8 ± 11.5	14.7 ± 11.2	17.3 ± 11.7	12.8 ± 11.1	n.s	14.3 ± 10.8	15.4 ± 12.5	n.s
Median (Range)	12 (1–57)	12 (1–49)	15 (3–45)	10 (1–37)		12 (1–57)	12(1–49)	
ICU /IMC treatment	171 (82.2)	90 (77.6)	25 (83.3)	48 (90.6)		109 (85.2)	62 (77.5)	
Mean ± SD	8.9 ± 9.8	8.5 ± 10	12.2 ± 8.8	7.31 ± 8.7	&0.022; #0.005; §n.s	8.4 ± 9.5	9.7 ± 10.3	n.s
Median (Range)	5.0 (1–46)	4 (1–46)	11 (1–31)	3.5 (1–37)		4 (1–46)	5 (1–41)	
Mechanical ventilation	44 (21.2)	19 (16.4)	11 (36.7)	12 (22.6)	&0.003; #n.s.; §n.s	28 (21.9)	16 (20)	n.s
For SE treatment	29 (13.9)	12 (10.3)	7 (23.3)	9 (17)		17 (13.3)	12 (15)	
For airway protection	15 (7.2)	7 (6)	4 (13.3)	3 (5.7)		11 (8.6)	4 (5)	
Limitation of therapy during SE	36 (17.3)	21 (18.1)	2 (6.7)	12 (22.6)	n.s	29 (22.7)	7 (8.8)	0.009
Medication								
ASM use prior SE	126 (60.6)	84 (72.4)	13 (43.3)	24 (45.3)	&0.006; #n.s.; §0.001	72 (56.3)	54 (67.5)	n.s
Mean number ± SD	1.6 ± 0.8	1.7 ± 0.9	1.5 ± 0.8	1.3 ± 0.5	n.s	1.5 ± 0.8	1.7 ± 0.8	n.s
Median number (Range)	1 (1–5)	1 (1–5)	1 (1–3)	1 (1–3)		1 (1–5)	1 (1–5)	
Number of ASMs used during SE								
Mean ± SD	3.6 ± 2.2	3.5 ± 2.1	3.6 ± 2.3	3.6 ± 2.5	n.s	3.6 ± 2.1	3.5 ± 2.4	n.s
Median (Range)	3 (1–10)	3(1–9)	3 (1–9)	3(1–10)		3 (1–10)	3(1–10)	
Number of ASMs at discharge								
Mean ± SD	2.2 ± 1.1	2.4 ± 1.1	2 ± 1	2 ± 1.1	n.s	2.1 ± 1	2.3 ± 1.2	n.s
Median (Range)	2 (1–5)	2 (1–5)	2 (1–5)	2 (1–4)		2 (1–5)	2 (1–5)	
Dexamethasone use at admission	64 (30.8)	43 (37)	2 (6.7)	18 (34)	& < 0.001; #0.002; §n.s	37 (28.9)	27 (33.8)	n.s
Mean ± SD dosage (mg)	6.4 ± 5.9	6.5 ± 6.2	5.5 ± 5	5.8 ± 5.4		7 ± 6.7	4.6 ± 2.3	
Median (Range) dosage (mg)	4 (0–24)	4 (0–24)	5.5 (2–9)	4(0–24)		4 (0–24)	4 (1–9)	
Dexamethasone use at discharge	101 (48.6)	63 (54.3)	3 (10)	30 (56.6)	& < 0.001; # < 0.001; §n.s	71 (55.5)	30 (37.5)	0.012
Mean ± SD dosage (mg)	7.8 ± 6.5	7.2 ± 6.6	7.7 ± 5.1	9.1 ± 6.7		9.2 ± 7.2	4.6 ± 2.3	
Median (Range) dosage (mg)	8 (1–48)	6 (1–48)	9 (2–12)	8(2–24)		8 (2–48)	4 (1–9)	
Tumor treatment								
Ongoing tumor spesific therapy at SE admission	61 (29.3)	44 (37.9)	0	15 (28.3)	& < 0.001; #0.001; §n.s	41 (32)	20 (25)	n.s
Temodal	27 (13.0)	26 (22.4)	0	0		20 (15.6)	7 (8.8)	
CCNU	7 (3.4)	7 (6)	0	0		4 (3.1)	3 (3.8)	
Other chemotherapy	15 (7.2)	3 (2.6)	0	11 (20.8)		9 (7)	6 (7.5)	
Immunotherapy	17 (8.2)	12 (10.3)	0	4 (7.5)		12 (9.4)	5 (6.3)	
Tumor treatment prior SE					n.t			n.t
Neurosurgery	87 (41.8)	39 (33.6)	9 (30)	31 (58.5)		64 (50)	23 (28.8)	
Radiation therapy	117 (56.3)	84 (72.4)	7 (23.3)	23 (43.4)		64 (50)	53 (66.3)	
Radiation	111 (53.4)	84 (72.4)	7 (23.3)	17 (32.1)		62 (48.4)	49 (61.3)	
Gamma knife	7 (3.4)	3 (2.6)	1 (3.3)	13 (24.5)		9 (7)	8 (10)	
Tumor specific therapy	113 (54.3)	67 (57.8)	1 (3.3)	38 (71.7)		69 (53.9)	44 (55)	
Radiation therapy < 6 months prior SE	53 (25.5)	43 (37.1)	0	9 (17)	& < 0.001; #0.01; §0.01	32 (25)	21 (26.3)	ns

p-values as pair-wise comparisons between tumor categories: & Adult-type diffuse gliomas vs. Intracranial extradural tumors; # Intracranial extradural tumors vs. Metastatic entities; § Adult-type diffuse gliomas vs. Metastatic entities, *n.s.* non significant, *n.t.* not tested, *ICU* Intensive care unit, *IMC* Intermediate Care, *SE* status epilepticus, *ASM* antiseizure medication

At admission, 64 (30.8%) patients were on dexamethasone, and this number increased to 101 (48.6%) at discharge. Patients with intracranial extradural tumors showed the lowest level of dexamethasone therapy (admission n = 2, 6.7%, discharge n = 3, 10%). In addition, patients without progression signs were more often receiving dexamethasone therapy at the time of admission (n = 27, 33.8%) than patients with progression signs (n = 37, 28.9%), but as expected, the increase in usage was more clear among patients with progression signs at discharge (with n = 71, 55.5% vs. without n = 30, 37.5%).

Prior to SE onset, 126 (60.6%) patients used an average of 1.6 ± 0.8 (median 1, range 1–5) ASMs, with the greatest usage by patients with adult-type diffuse gliomas (n = 84, 72.4%) compared to the other two tumor categories. During the SE period, the average number of treatment steps with an ASM was 3.6 ± 2.2 (median 3, range 1–10), and patients were discharged with an increased mean number of ASMs (2.2 ± 1.1, median 2, range 1–5, p < 0.001); a detailed description of the treatment steps is presented in Additional file 1: Table S2.

The mean length of stay (LOS) in hospital was 14.8 ± 11.5 days (median 12, range 1–57 days). Of all patients, 171 (82.2%) were admitted to the intensive care unit (ICU) or intermediate care (IMC) department and 44 (21.2%) required mechanical ventilation. The mean LOS in the ICU/IMC was 8.9 ± 9.8 days (median 5, range 1–46), while patients with intracranial extradural tumors had hospital (17.3 ± 11.7, median 15, range 3–45 days) and ICU/IMC (12.2 ± 8.8, median 11, range 1–31 days) stays that were significantly longer and required mechanical ventilation more often than patients from both other tumor categories. Treatments or administered therapies were limited during the SE event in 36 (17.3%) patients, with less limitations for those with intracranial extradural tumors (n = 2, 6.7%); however, significantly reduced levels of therapy were noted in patients with signs of tumor progression (n = 29, 22.7%). The detailed information on inpatient stay, medications, and tumor treatments of the SE patients is presented in Table 3.

#### Outcome

**Fig. 1:**
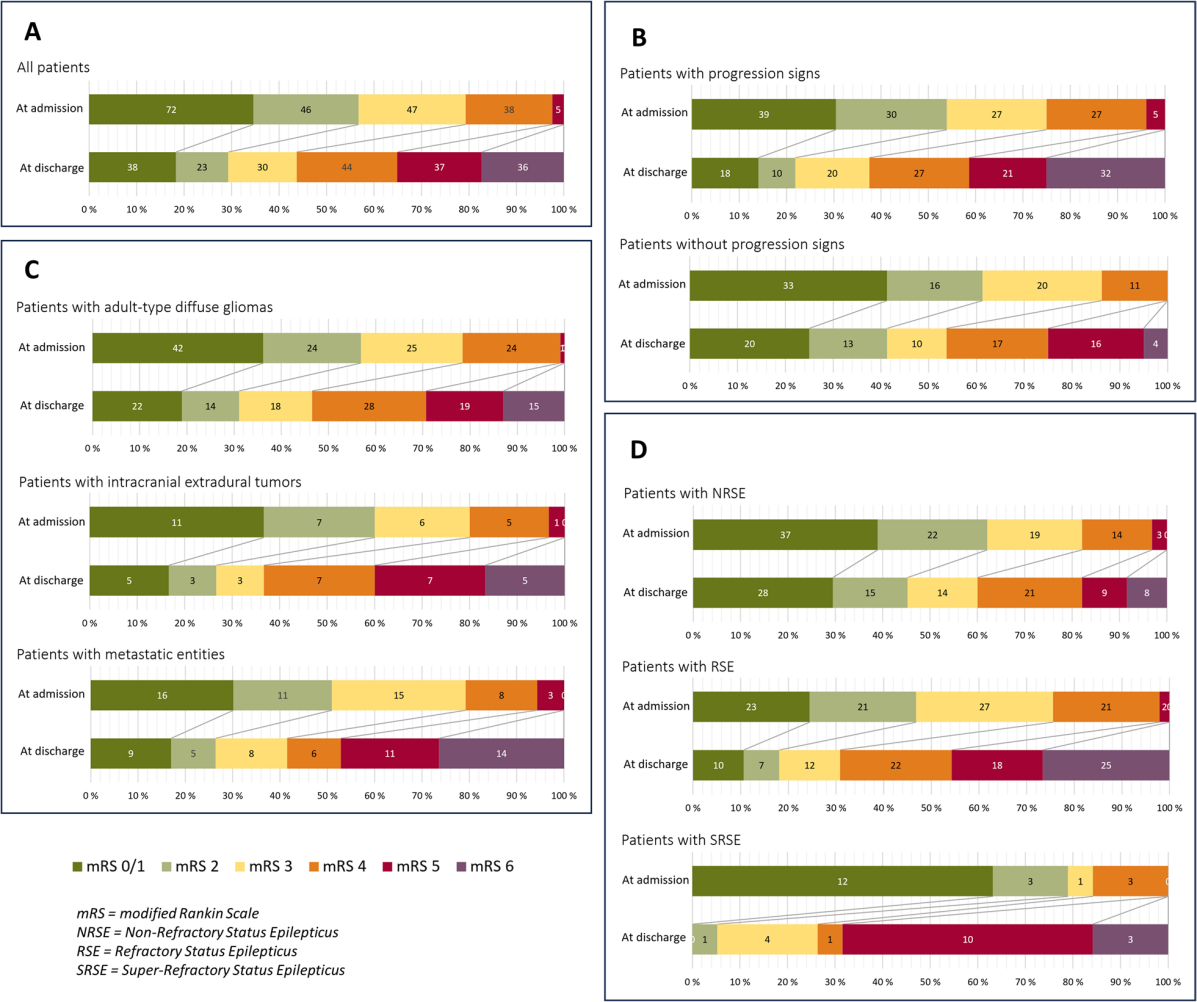
Functional status of all patients measured by modified Rankin Scale before and after SE (**A**) stratified by tumor progression signs (**B**), tumor category (**C**), and SE refractoriness (**D**)

In total, 61 (29.4%) patients were discharged with good functional status (mRS 0–2), 111 (53.3%) had poor functional status (mRS 3–5), and 36 (17.3%) patients were deceased. The functional status at discharge did not differ significantly between the tumor categories; however, a clear difference was observed depending on the presence of the radiological criteria of tumor progression, with only 28 (21.9%) of the patients with progression presenting with good functional outcomes compared to 33 (41.3%) of those without progression. A clear shift to a poorer functional status at discharge compared to that at admission was significant for all patients (p < 0.001) and all subgroups (tumor category, progression signs, and refractoriness; all p-values < 0.001). The mRS distribution of the subgroups is shown in Fig. 1. The mortality at discharge was higher among patients with metastatic entities (n = 14, 26.4%) than those with adult-type diffuse gliomas, and higher among patients with progression signs (n = 32, 25%) than those without (n = 4, 5%) these signs.

**Fig. 2:**
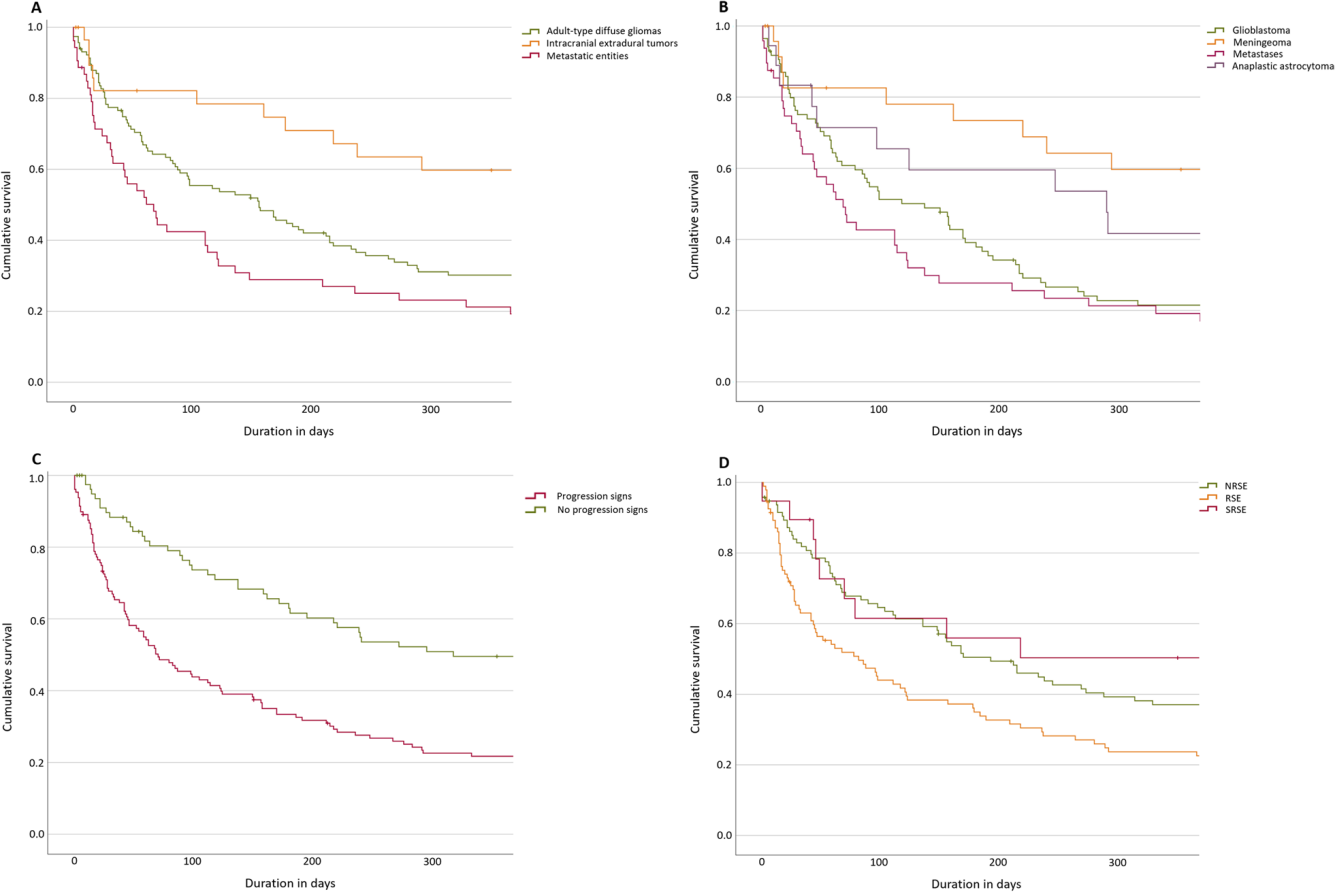
Long-term outcomes depicted by Kaplan-Maier curves stratified by tumor category (**A**, **B**), tumor progression signs (**C**), and SE refractoriness (**D**)

The long-term patient survival at one year is depicted using Kaplan–Meier curves in Fig. 2. Survival among patients with intracranial extradural tumors was higher than that for patients with adult-type diffuse gliomas (log-rank p-value 0.002) and metastatic entities (log-rank p-value < 0.001) (Fig. 2a). There was no difference between the results for the latter two groups (log-rank p-value 0.059). Figure 2b shows the survival of patients with meningiomas, glioblastomas, and solid metastases. Patients with signs of tumor progression had significantly poorer survival rates than those without signs of progression (log-rank p-value < 0.001) (Fig. 2c). Refractoriness is depicted in Fig. 2d, whereby patients with NRSE showed better survival rates than patients with RSE (SRSE not included, log-rank p-value 0.020), while no statistical difference was identified between NRSE and SRSE patients (log-rank p-value 0.512) or between RSE and SRSE patients (log-rank p-value 0.066). There was no difference between those with a previous history of epilepsy or SE compared to those with new-onset SE (log-rank p-value 0.456, Kaplan-Meier curves not shown).

## Discussion

This retrospective, multicenter study described the clinical characteristics, treatment, and outcomes of 208 SE patients with brain tumors and metastases using the largest identified cohort published to date. The results indicated that the SE event caused a clear decline in the functional status (mRS) of the patients, which was independent of tumor type, radiological progression, and SE refractory level. Less than half of the patients survived for a year after the SE event, and considerable mortality was observed among the patients with nonmalignant intracranial extradural tumors. The SRSE cases presented better survival rates than those of the less refractory cases at one year after SE. The definition of SRSE requires anesthesia and only patients with a better outcome are likely to be considered for anesthesia. In principle, refractoriness could also be defined according to the duration of SE and number of therapies; this is currently being discussed and researched [27–31].

The existing literature regarding SE in patients with brain tumors is scarce [9, 15], despite brain tumors presenting a high risk for the development of SE and accounting for approximately 10% of all SE cases [10, 32]. A limited number of studies have focused on this specific patient group, and the majority of these were retrospective, involved only a single center, and consisted of small patient cohorts. Our literature review identified seven original studies [14, 16, 33–37] and two reviews [9, 15], the details of which are presented in Table 4. These studies indicated that SE patients with brain tumors averaged 55–68 years of age and mainly included males. The data from the present cohort were concordant with those of the previous studies, although the sex distribution differed significantly between tumor categories, as patients with adult-type diffuse gliomas were more likely to be male, while those from other groups were more often female. In addition, patients with intracranial extradural tumors were significantly older than the patients from the other groups.

**Table 4:** Summary of published studies on status epilepticus cases caused by brain tumors and metastases

Author	Year	Study design	Setting	Study period	SE type	Tumor type	Patients (N)	Age (median)	Male	In-hospital mortality	30d mort	RSE/SRSE
Current study: Rickel	2024	Retrospective	Multicenter	2011–2017	SE	Brain tumors and metastases	208	62	51%	17.3%	24%	54.3%
Kaneoka	2023	Retrospective	Single center	2013–2019	NCSE	Gliomas	5	57**	58.3%	–	–	–
Vilaseca-Jolonch	2020	Prospective, observational	Single center	2011–2019	SE	Brain tumors	82	61.6	58.5%	17.1%	–	52.5%
Giovannini	2019	Prospective, observational	Single center	2013–2019	SE	Gliomas	26	68	58%	–	4%	12%
Fox	2019	Retrospective	Single center	2006–2018	SE	Brain metastasis	19	63.9**	47.4%	5.3%	31.6%	57.9%
Knudsen-Baas	2016	Prospective, observational	Multicenter	2008–2014	SE	Gliomas	20	55	80%	0%	30%	26%
Goonawardena	2015	Review	–	1955–2013	SE	Brain tumors	–	–	–	–	12–50%	12–18%
Marcuse	2014	Retrospective	Single center	2009–2012	NCSE	Brain tumors	24	62**	50_-_%	0%	48% ***	–
Arik	2014	Review (14 sudies*)		1994–2009	SE	Brain tumors	417	–		17.2%		–
Cavaliere	2006	Retrospective	Single center	1995–2002	SE	Brain tumors	35	58	65.7%	–	23%	–

*Review of literature regarding outcome of status epilepticus. **Age presented as Mean. ***Mortality at 2 months, *NCSE* non-convulsive status epilepticus, *SE* status epilepticus

Adult-type diffuse gliomas and intracranial extradural tumors accounted for the vast majority of primary brain tumors in this study (70.2%), while 29.9% of the patients had metastatic entities or other tumors. The proportion of adult-type diffuse gliomas (55.8%) was comparable with the results of previously reported SE cohorts that indicated 41.5–65.7% for malignant brain tumors or gliomas (high-grade and low-grade gliomas) [18, 34, 37]. Similarly, the intracranial extradural tumor (14.4%) and metastatic entity (25.5%) percentages corroborated those of previous studies, which reported 14.3–25% of benign brain tumors/meningiomas and 26.8%–34.4% of metastatic tumors [16, 34, 37]. These findings were expected, as a higher frequency of seizures and SE events occurs among patients with primary brain tumors compared to those with metastasis [5, 16] and a higher risk of SE is associated with increasing WHO grades [9, 14, 36, 38].

Arik et al. conducted a systematic review that considered SE outcomes and found that SE events caused by brain tumors have higher in-hospital mortalities than non-anoxic SE caused by other factors (17.2% vs. 6%) [15]. Goonawardena et al. compared tumor-associated SE (TASE) to SE in the general population and tumor-associated epilepsy (TAE) in a systematic review and found that the 30-day mortality of TASE ranged between 12 and 50%, which was similar to or higher than the mortality percentage of the general population [9]. In the present cohort, the overall mortality at discharge (17.3%) and at 30 days (24%) was comparable to the 30 days mortality range estimated by Goonawardena et al. [9] and the 17.1% in-hospital mortality identified in a prospective cohort of 82 patients published by Vilaseca-Jolonch et al. [34]

Our data showed that the highest in-hospital mortality rate occurred among patients with metastatic entities (26.4%) and that this value was significantly higher than that of patients with adult-type diffuse gliomas. Higher short-term mortalities and poorer outcomes in patients with brain metastases compared those with to primary brain tumors are consistent with the current literature [16, 34, 37]. High-grade gliomas have shown greater 30-day mortality rates (20%) than low-grade gliomas with no mortality [36]. In our cohort, patients with adult-type diffuse gliomas had an in-hospital mortality rate of 13%; however, a clear increase in mortality (up to 70%) was noticed during the first year after discharge. In particular, the survival of glioblastoma patients was similar to that of metastatic entity patients at one year and distinctively lower than that of patients with other types of glioma or meningioma. In this regard, the long-term mortality of TASE has been related to the natural prognosis of tumor types [14].

Radiological progression was present in the majority of SE patients in the cohort (61%). Previous literature regarding the occurrence of SE concomitant with progression is inconsistent, as no difference was observed between patients with stable tumors and those with tumor progression in terms of the occurrence of SE in the study by Knudsen-Baas et al. [36], although the occurrence of TASE has been associated with tumor progression [9]. Progressing brain lesions and tumor recurrences are associated with a risk of mortality within 2 months of the SE [37]. Similarly, tumor progression at the onset of SE has been associated with long-term mortality, with up to 73.3% of the patients dying due to tumor progression during the median 10.5-month follow-up period [34]. These associations were confirmed in our cohort. Radiological signs of CNS-related tumor progression at admission were most prevalent in patients with metastatic entities and least evident in those with intracranial extradural tumors, which could partly explain the poor outcomes of the former group. Dexamethasone use at admission was more frequent among patients without signs of progression but naturally increased among patients showing signs of progression, which accounts for the significant difference between the groups.

Giovannini et al. identified no increases in mortality or morbidity after a TASE episode among glioma patients [14]. Another study reported that 30% of patients acquired new neurological deficits after glioma-associated SE; however, this finding was suggested to be related to tumor progression as an etiology of SE [36]. Our study showed a statistically significant decline in the functional status of all patients during the SE treatment period. SRSE showed the largest overall decline, which is in line with the results of other studies [39]. In addition, patients with intracranial extradural tumors experienced a clear decline in functional status. The pre-SE functional status did not differ between the subgroups; however, the status at discharge was significantly poorer in patients with signs of tumor progression compared to those without progression signs.

Previous studies have reported tumor-associated SE refractoriness ranging from 12 to 57.9% [9, 14–16, 33–37]. Reviews have indicated that TASE tends to be more responsive than SE to first- and second-line ASMs in the general population, with refractoriness evident in only 12–18% of cases [9]. Furthermore, TASE appears to have a shorter duration than SE in the general population (153 min vs. 174 min) [15]. The present study showed a markedly high proportion of refractoriness (54.3%), which is comparable with the 52.5% refractoriness level found in another cohort study [34]. In addition, refractoriness was not related to tumor category or the signs of progression, which corroborates the results of a previous study [36]. Although SRSE has been associated with poor prognoses in SE patients, the SRSE cases identified here showed better outcomes than those of the less refractory cases at one year [29, 40, 41]. This result could be partly explained by the variation in SE treatment approaches based on the overall prognosis of the patients; thus, patients with better tumor prognoses might be offered more therapy options, such as intubation and anesthesia treatment, whereas patients with poorer overall prognoses are more likely to be administered ASM treatment without the use of anesthesia. This study showed that therapy limitations were more common in patients with signs of tumor progression.

The majority of patients in this study had a previous history of seizures (61.1%), whereas other cohorts showed slightly lower proportions (35–56.3%) [14, 16, 34–36]. This result was more prominent in patients with adult-type diffuse gliomas and those without progression signs. A new brain tumor diagnosis was made in every fourth patient at admission. The lowest percentage (15.8%, range 15.8–54%) of newly diagnosed tumors at SE onset reported in the literature occurred in a study focusing on SE in patients with brain metastasis, while our study determined that a new tumor was usually diagnosed in patients with metastatic entities or signs of progression. The overall time between tumor diagnoses and SE (2.3 years) was considerably longer than the 60 days to 4 months reported in other studies [14, 35]. The longest interval was found among patients with intracranial extradural tumors and the shortest was identified among those with metastatic entities and tumors with progression signs. Our findings confirm the overall supposition that SE occurs later in the disease course or prior to tumor progression [9, 42].

The median LOS in hospital (12 days) was comparable to that of TASE patients in another study (9 days) [34]. However, the LOS value was in the upper range of the reported median of 5–14 days among general SE patients [29, 41, 43, 44]. The present study is the first identified report that determined the ICU LOS in the patient group (median 5 days), which was revealed to be slightly higher than the LOS for general SE patients (3 days) [29]. Similar to the 22% reported by Marcuse, 21.2% of the patients required mechanical ventilation [37].

Finally, intracranial extradural tumors were the predominant tumors in all categories studied. The associated patients differed significantly from those with adult-type diffuse gliomas, as they presented more frequently with SRSE, required additional mechanical ventilation, and endured longer ICU stays. In addition, intracranial extradural tumor patients showed less frequent tumor progression signs than patients with other tumors and were administered less dexamethasone treatments than adult-type diffuse glioma patients. However, the decline in the functional status of these patients was clear during the SE and the mortality at one year reached 36.7%. The older age of these patients may partly explain the poor outcomes; however, the direct effect of SE and related treatments cannot be discounted.

### Limitations

This study is limited by its retrospective design. However, the multicenter approach covering a study period of over seven years and the considerable amount of material reviewed increase the reliability of the results.

Direct comparisons of the results with prior studies were compromised by the heterogeneity in study design and differences in tumor categorization. The recent increase in the biomolecular understanding of brain tumors has changed the field, but since this retrospective study aimed for a comprehensive cohort, gliomas were included that were diagnosed according to former WHO CNS tumor classification guidelines. Causes of death were not assessed; therefore, it was not possible to differentiate whether patients died as a direct consequence of SE, related complications, or withdrawal of care. Furthermore, we provided only descriptive data on different tumor therapies such as neurosurgery, radiation, and chemo- and immunotherapies, and further studies with each tumor entity are required to determine the correlations among different therapies and their benefits or risks related to seizure and SE occurrence [45]. In addition, future studies focusing on specific tumor entities should address the resection extent of diffuse gliomas and metastatic burden in patients with metastatic tumors.

## Conclusion

SE occurrence in patients with brain tumors contributes significantly to an immediate decline in functional status, regardless of the type of tumor (malignant or non-malignant), presence or absence of tumor progression, and refractoriness of the SE episode. Therefore, effective prevention of SE events is imperative for preserving and maintaining functional status in individuals with brain tumors.

### Supplementary Information

**Additional file 1. Table S1.** Tumor localization; **Table S2.** Treatment characteristics with antiseizure medications (ASMs) and anesthetics.

## Data Availability

The datasets used and analyzed during the current study are available from the corresponding author on reasonable request.
