# Supplementary material for: Status epilepticus in patients with brain tumors and metastases: A multicenter cohort study of 208 patients and literature review
Source: Neurol Res Pract. 2024 Apr 4;6:19. doi: 10.1186/s42466-024-00314-7 (PMC10993483; doi:10.1186/s42466-024-00314-7)
Supplement: Supplementary file 1 — Additional file 1. Table S1. Tumor localization; Table S2. Treatment characteristics with antiseizure medications (ASMs) and anesthetics. [file 42466_2024_314_MOESM1_ESM.docx]

**Supplementary Tables**

Table S1. Tumor localization

| Variables |  | N (%) |
| --- | --- | --- |
| All patients |  | 208 (100) |
| **Hemisphere** |  |  |
|  | Right | 96 (46.2) |
|  | Left | 74 (35.6) |
|  | Bihemispheric | 38 (18.3) |
| **Cerebral region** | |  |
|  | Frontal lobe | 94 (45.2) |
|  | Temporal lobe | 66 (31.7) |
|  | Parietal lobe | 61 (29.3) |
|  | Occipital lobe | 20 (9.6) |
|  | Cerebellum | 9 (4.3) |
|  | Diencephalon | 7 (3.4) |
|  | Brainstem | 1 (0.5) |
|  | Multilocular | 15 (7.2) |

Table S2. Treatment characteristics with ASMs and anesthetics

|  | **All patients** | **Bolus (mg)** | | | **Maintenance dose (mg)** | | |
| --- | --- | --- | --- | --- | --- | --- | --- |
| **Benzodiazepines** | **N (%)** | **Mean** | **Median** | **Range** | **Mean** | **Median** | **Range** |
| Midazolam | 61 (29.3) | 7 | 5 | 1-25 | 8 | 5 | 1-20 |
| Diazepam | 12 (5.8) | 16.25 | 12.5 | 5-40 | 18.75 | 12.5 | 10-40 |
| Lorazepam | 152 (73.1) | 2 | 2 | 0.5-8 | 3 | 3 | 1-15 |
| Clonazepam | 6 (2.9) | 1 | 1 | 0 | 1.75 | 1-5 | 1-3 |
| **Intravenous ASM** |  |  |  |  |  |  |  |
| Levetiracetam | 158 (76.0) | 1365 | 1000 | 250-4000 | 3287 | 4000 | 500-8000 |
| Valproate | 97 (46.6) | 1026 | 800 | 200-3000 | 2525 | 2400 | 200-6600 |
| Lacosamide | 95 (45.7) | 164 | 200 | 50-400 | 363 | 400 | 100-800 |
| Phenytoin | 35 (16.8) | 652 | 750 | 100-1500 | 767 | 750 | 300-1500 |
| Brivaracetam | 4 (1.9) | 112.5 | 112.5 | 25-250 | 250 | 250 | 100-400 |
| **Oral ASM** |  |  |  |  |  |  |  |
| Clobazam | 27 (13.0) | 6 | 5 | 5-15 | 14.5 | 15 | 5-30 |
| Topiramate | 19 (9.1) | 163 | 100 | 25-500 | 465 | 400 | 100-900 |
| Perampanel | 10 (4.8) | 7 | 6 | 3-12 | 11 | 12 | 6-12 |
| Zonisamide | 9 (4.3) | 108 | 100 | 25-200 | 256 | 200 | 50-600 |
| Gabapentin | 4 (1.9) | 250 | 300 | 100-300 | 900 | 900 | 0 |
| Lamotrigine | 4 (1.9) | 62.5 | 37.5 | 25-150 | 150 | 100 | 50-300 |
| Oxcarbazepine | 3 (1.4) | 350 | 300 | 150-600 | 1250 | 900 | 450-2400 |
| Carbamazepine | 2 (1.0) | 600 | 600 |  | 1200 | 1200 |  |
| Stiripentol | 1 (0.5) | 500 | 500 |  | 1000 | 1000 |  |
| **Anesthetics used for burst-suppression** |  |  |  |  |  |  |  |
| Propofol | 26 (12.5) |  |  |  | 4358 | 4740 | 700-8640 |
| Midazolam | 18 (8.7) |  |  |  | 597 | 600 | 330-1000 |
| Ketamine | 9 (4.3) |  |  |  | 2831 | 2300 | 30-9600 |
| Thiopental | 6 (2.9) |  |  |  | 4010 | 2100 | 400-11050 |
